# Association of Preconception Thyrotropin Levels With Fecundability and Risk of Spontaneous Abortion in China

**DOI:** 10.1001/jamanetworkopen.2022.28892

**Published:** 2022-08-31

**Authors:** Ying Yang, Tonglei Guo, Jinrong Fu, Jun Zhao, Yuanyuan Wang, Yuan He, Zuoqi Peng, Ya Zhang, Hongguang Zhang, Yue Zhang, Qiaomei Wang, Haiping Shen, Yiping Zhang, Donghai Yan, Xu Ma, Haixia Guan

**Affiliations:** 1 National Research Institute for Family Planning, National Human Genetic Resource Center, Beijing, China; 2 Graduate School of Peking Union Medical College, Dongdan Santiao, Beijing, China; 3 Department of Endocrinology, Guangdong Provincial People's Hospital, Guangdong Academy of Medical Sciences, Guangzhou, China; 4 Department of Endocrinology and Metabolism, The First Hospital of China Medical University, Shenyang, China; 5 Department of Maternal and Child Health, National Health Commission of the People’s Republic of China, Beijing, China

## Abstract

**Importance:**

Abundant evidence suggests thyroid dysfunction is associated with adverse pregnancy outcomes. However, associations of preconception thyrotropin levels outside of reference range with reproductive health outcomes are not well characterized.

**Objective:**

To evaluate the associations of preconception thyrotropin levels with time to pregnancy (TTP) and risk of spontaneous abortion (SA).

**Design, Setting, and Participants:**

This population-based cohort study used data from the Chinese National Free Prepregnancy Checkups Project. Female participants aged 20 to 49 years who were trying to conceive were enrolled between January 1, 2013, and December 31, 2016, for the analysis of TTP or SA. Data were analyzed between August 1, 2020, and July 5, 2021.

**Exposures:**

Levels of thyrotropin within 1 year prior to pregnancy.

**Main Outcomes and Measures:**

The main outcomes were TTP, assessed using hazard ratios (HRs), and SA, assessed using odds ratios (ORs), according to preconception thyrotropin levels. Thyrotropin dose-response associations were assessed using restricted cubic spline regression.

**Results:**

Among 11 194 002 female participants (mean [SD] age, 27.56 [5.10] years) in the TTP cohort, 4 739 421 (42.34%) participants became pregnant within 1 year. Both low and high preconception thyrotropin levels were associated with delayed TTP compared with thyrotropin levels of 0.37 to 2.49 mIU/L (thyrotropin <0.10 mIU/L: HR, 0.90; 95% CI, 0.89-0.92; thyrotropin 4.88-9.99 mIU/L: HR, 0.86; 95% CI, 0.86-0.87; thyrotropin ≥10.00 mIU/L: HR, 0.78; 95% CI, 0.77-0.79). In the SA analysis cohort including 4 678 679 pregnancies, 108 064 SA events (2.31%) were documented. High thyrotropin groups showed an increased risk of SA compared with the group with thyrotropin levels of 0.37 to 2.49 mIU/L (thyrotropin 4.88-9.99 mIU/L: OR, 1.33; 95% CI, 1.28-1.38; thyrotropin ≥10.00 mIU/L: OR, 1.25; 95% CI, 1.14-1.36). Preconception thyrotropin levels showed an inverted J-shaped dose-response association with TTP (χ^2^ = 311.29; nonlinear *P* < .001) and a J-shaped dose-response association with SA (χ^2^ = 58.29; nonlinear *P* < .001).

**Conclusions and Relevance:**

In this cohort study, preconception thyrotropin levels outside of reference range were associated with increased risk of reduced fecundity and SA. These findings may provide insights for the implementation of preconception thyroid function screening and the design of future levothyroxine supplementation trials.

## Key Points

**Question:**

Are preconception thyrotropin levels associated with fecundability and risk of spontaneous abortion?

**Findings:**

In this cohort study of 11 194 002 participants in China, participants with preconception thyrotropin levels outside reference range were significantly more likely to experience delayed time to pregnancy and increased risk of spontaneous abortion. A preconception thyrotropin level of 0.37 to 2.49 mIU/L was associated with the lowest risk of these unfavorable outcomes.

**Meaning:**

The findings of this study suggest that interventional studies investigating the benefits of preconception thyroid function screening and levothyroxine supplementation are warranted.

## Teaser

This cohort study evaluates associations of preconception thyrotropin levels with time to pregnancy and risk of spontaneous abortion in a large population-based cohort in China.

## Introduction

Infertility affects approximately 9% of couples of reproductive age worldwide.^1^ Even among people who have conceived successfully, 8% to 30% are at risk of spontaneous abortion (SA), which is one of the most common complications during pregnancy.^2^ In China, the prevalence of female infertility is estimated to be 15% to 20%, and approximately 25% of couples have trouble conceiving or sustaining pregnancy.^3^ Hence, identifying the risk factors that may disrupt reproductive health and lead to infertility is needed.^4^

Thyroid dysfunction is closely intertwined with hypothalamic-pituitary-ovarian axis.^5^ Both hypothyroidism and hyperthyroidism are associated with menstrual irregularity and implantation failure.^6,7^ Even subclinical hypothyroidism (SCH) has been reported to be a risk factor for ovulatory dysfunction or tubal disease.^8,9^ A prospective, randomized trial of 64 patients with SCH undergoing infertility treatment showed levothyroxine treatment significantly decreased miscarriage rate by 33.3% and increased live birth rate by 28.1%.^10^ In addition to maternal reproductive health, thyroid hormone is also essential for placental and fetal development. Increasing evidence suggests that the association of thyroid dysfunction with pregnancy outcomes initiates as early as during the periconception period.^11,12^ As thyroid physiology changes during pregnancy, mild thyroid hormone deficiency may progress to overt hypothyroidism, which can contribute to adverse obstetric outcomes, including preterm birth, miscarriage, and neurocognitive disorders in offspring.^13^

However, the evidence on the impact of preconception thyroid function on time to pregnancy and fecundability are limited and primarily investigated among patients undergoing infertility treatment or with a history of pregnancy loss.^14,15^ Also, in the absence of a universal recommended thyrotropin range for individuals planning for pregnancy, screening of thyroid function is not consistently performed by clinicians.^16^ In this study, we investigated the associations of preconception maternal thyrotropin levels with fecundability and risk of SA in a population-based cohort.

## Methods

This cohort study was approved by the Institutional Research Review Board at the National Research Institute for Family Planning in Beijing, China. Every participant provided written informed consent before enrollment. The reporting of this study followed the Strengthening the Reporting of Observational Studies in Epidemiology (STROBE) reporting guideline.

### Study Population

**Figure 1.**
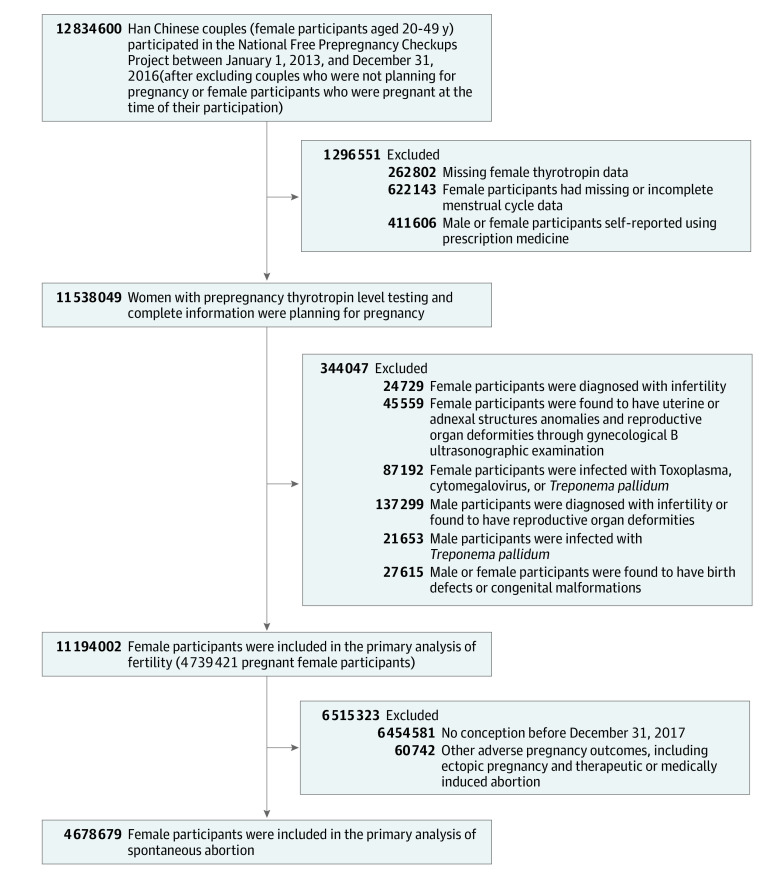
Flowchart of the Study Cohort Selection Criteria

The National Free Prepregnancy Checkups Project (NFPCP) is a national preconception health care service launched by the National Health Commission and the Ministry of Finance of the People’s Republic of China in 2010. It aims to provide free preconception health examinations, counselling, and follow-up of pregnancy outcomes for reproductive-aged (20-49 years) couples who plan to conceive. More detailed information about the design, organization, and implementation of the NFPCP has been previously reported.^17,18,19^ Because Han Chinese female participants account for more than 90% of the total population in the NFPCP and there may be differences in preconception thyrotropin levels among female participants by ethnicity, our study included only Han Chinese female participants. Trained interviewers or health care staff collected participants’ ethnicity by their identification cards in local maternal and child service centers during the preconception health examination period. A total of 12 834 600 Han Chinese female participants aged 20 to 49 years and their spouses enrolled in NFPCP between January 1, 2013, and December 31, 2016, from 2753 counties in 31 provinces throughout China. The couples confirmed that they desired immediate conception, and the female participants were not pregnant at the time of their enrollment. A flowchart of the exclusion criteria for the study population is provided in Figure 1. All participants completed follow-up for their pregnancy outcome by December 31, 2017. Data were analyzed between August 1, 2020, and July 5, 2021.

### Data Collection and Outcomes

For each participant in the NFPCP, different types of data were collected and recorded in 3 main stages: preconception health examinations, early pregnancy follow-up, and pregnancy outcome follow-up (eAppendix 1 in the Supplement). Time to pregnancy (TTP) in cycles was defined and calculated by the following formula: TTP = (date of the last menstrual period [in pregnant participants] or date of the most recent follow-up [nonpregnant participants] − date of enrollment / mean menstrual cycle length) + 1.^20,21^ A participant’s self-reported mean menstrual cycle length was based on their menstrual history in the recent 6 months. In our study, a regular menstrual cycle was defined as a cycle with an intermenstrual interval between 21 and 35 days, and the variation of cycle length from 1 period to another was no more than 7 days.^22^ SA was defined as fetal death or pregnancy loss occurring before the 28th week of gestation.

### Serum Thyrotropin Measurements

During the preconception examination, venous blood samples after at least 8 hours of fasting were collected from each participant and immediately sent to the laboratories of local maternal and child service centers where the blood sample was separated and stored at −30 °C. Maternal serum thyrotropin levels were measured according to the National Guide to Clinical Laboratory Procedures. Due to the magnitude of the NFPCP, it was impractical to use a single type of thyrotropin detection kit or method across all local laboratories of 2753 counties in 31 provinces. Each county consistently used a uniform type of thyrotropin assay, either electrochemiluminescence immunoassay or enzyme-linked immunosorbent assay kits. In a previous analysis of the same database, a population-specific thyrotropin reference range was established.^12^ The reference range for preconception thyrotropin levels was defined with a median of 0.37 (95th percentile, 1.66-4.88) mIU/L.

In addition, preconception thyrotropin values were converted to multiples of the median (MOM) for further analyses to overcome the systematic differences or variability among various local laboratories or assays. Thyrotropin MOM value was calculated by dividing the participant’s thyrotropin value by the median thyrotropin value in the reference population of the county where they registered to participate in the preconception examination.

### Statistical Analysis

Continuous variables with normal distribution were expressed as mean and SD, and nonnormally distributed variables were expressed as median and IQR. Categorical variables were expressed as numbers (percentages) for baseline characteristics. The Mann-Whitney *U* test was used to compare the difference of thyrotropin levels among the different groups.

Hazard ratios (HRs) and 95% CIs were estimated by Cox proportional hazards regression models to examine the association of preconception thyrotropin levels and thyrotropin MOM values with TTP. An HR of less than 1 indicates a reduction in fecundability or a longer TTP, whereas an HR of greater than 1 indicates a shorter TTP. We fitted 2 Cox proportional hazards regression models with a priori selected covariates. The crude model (model 1) only adjusted for a participant’s age at last menstrual period. The multivariable model (model 2) further adjusted for characteristics of female participants and potential factors associated with fecundability: preconception body mass index (BMI); education, area of residence, alcohol drinking, smoking, passive smoking, hypertension, type 2 diabetes, history of thyroid disease, reproductive tract infections, hepatitis B virus surface antigen–positivity status, age at menarche, menstrual cycle length, and menstrual period length.

The risk of SA was estimated by logistic regression models to examine the association of preconception thyrotropin levels and with thyrotropin MOM values with SA. We fitted 2 logistic regression models with a priori selected covariates. The crude model (model 1) adjusted for participant’s age at the last menstrual period. In the multivariable model (model 2), we additionally adjusted for BMI, education, area of residence, alcohol drinking, smoking, passive smoking, hypertension, diabetes, history of thyroid disease, and adverse pregnancy outcomes. Detailed definitions of these variables can be found in eAppendix 2 in the Supplement.

Preconception thyrotropin levels were stratified into 6 groups in all the models: group 1, less than 0.10 mIU/L; group 2, 0.10 to 0.36 mIU/L; group 3, 0.37 to 2.49 mIU/L (reference group); group 4, 2.50 to 4.87 mIU/L; group 5, 4.88 to 9.99 mIU/L; and group 6, ≥10.00 mIU/L. We did not stratify participants according to thyroid autoimmunity status because measurement of thyroid autoantibodies was not a routine test in China. Sensitivity analyses were conducted by excluding participants with irregular menstrual cycles or with missing data on baseline characteristics.

The dose-response associations of maternal preconception thyrotropin levels and thyrotropin MOM values with outcomes were assessed using restricted cubic spline. Five knots at the 5th, 25th, 50th, 75th, and 95th percentiles of preconception thyrotropin levels were used in plotted smooth curves, and the nonlinearity of the dose-response association was tested by Wald statistics.^23^ Covariates in dose-response association analyses models were same as the covariates in the previous Cox proportional hazards regression model 2 for TTP or logistic regression model 2 for SA. In addition, subgroup analysis was performed according to gravidity or history of adverse pregnancy outcomes.

Statistical analysis was performed using R software version 4.0.3 (R Project for Statistical Computing) with the analysis packages survival, version 3.2-7; forestplot, version 1.1.0; data.table 1.13.6; rms, version 6.1-1; ggplot2, version 3.3.3 and speedglm, version 0.3-3. All statistical tests were 2-sided, and *P* < .01 was considered statistically significant. Data were analyzed from August 1, 2020, to July 5, 2021.

## Results

### Cohort Characteristics

A total of 11 194 002 participants (mean [SD] age. 27.56 [5.10] years; 4 739 421 [42.34%] became pregnant) who completed follow-up between 2013 to 2017 in the NFPCP were included in the analysis of fecundability (Figure 1). Meanwhile, 4 678 679 participants (mean [SD] age, 26.54 [4.19] years; 108 064 [2.31%] SA events) who successfully conceived and completed follow-up for their pregnancy outcome by December 31, 2017, were included in the analysis of SA (Figure 1). Characteristics of the participants in fecundability analysis are presented in the Table, and characteristics of participants in the SA analysis are presented in eTable 1 in the Supplement.

**Table.** Characteristics of the Participants in Fecundability Analysis

Characteristics	Characteristics, No. (%)	
	Maternal (n = 11 194 002)	Paternal (n = 11 194 002)
Age at baseline, y^a^		
20-24.9	3 907 059 (34.90)	2 461 550 (22.00)
25-29.9	4 563 162 (40.76)	4 872 792 (43.53)
30-34.9	1 640 656 (14.66)	2 197 390 (19.63)
35-39.9	718 086 (6.42)	998 254 (8.92)
≥40	365 039 (3.26)	617 755 (5.52)
Missing data	NA	46 261 (0.41)
BMI		
<18.5	1 400 912 (12.51)	445 134 (3.98)
18.5-23.9	7 846 294 (70.09)	6 871 985 (61.39)
24.0-27.9	1 529 807 (13.67)	3 021 343 (26.99)
≥28.0	389 750 (3.48)	822 283 (7.35)
Missing data	27 239 (0.24)	33 257 (0.30)
Thyrotropin level, mIU/L		
<0.36	265 288 (2.37)	NA
0.36-4.87	10 569 225 (94.42)	NA
≥4.88	359 489 (3.21)	NA
Education		
≥High school	1 879 871 (16.79)	1 918 103 (17.14)
≤Primary school	9 118 609 (81.46)	9 091 325 (81.22)
Missing data	195 522 (1.75)	184 574 (1.65)
Residence		
Rural	10 127 391 (90.47)	9972719 (89.09)
Urban	1 066 086 (9.52)	1220714 (10.91)
Missing data	525 (<0.01)	569 (<0.01)
Alcohol consumption		
Yes	320 448 (2.86)	3 110 091 (27.78)
No	10 841 341 (96.85)	8 057 658 (71.98)
Missing data	32 213 (0.29)	26 253 (0.23)
Smoking status		
Yes	30 299 (0.27)	3 104 999 (27.74)
No	11 140 867 (99.53)	8 068 566 (72.08)
Missing data	22 836 (0.20)	20 437 (0.18)
Second-hand smoke		
Yes	1 206 274 (10.78)	2 538 541 (22.68)
No	9 964 629 (89.02)	8 621 352 (77.02)
Missing data	23 099 (0.21)	34 109 (0.30)
Hypertension		
Yes	236 728 (2.11)	594 265 (5.30)
No	10 905 305 (97.42)	10 541 115 (94.17)
Missing data	55 250 (0.49)	58 622 (0.52)
Diabetes		
Yes	142 026 (1.27)	NA
No	10 999 591 (98.26)	NA
Missing data	52 385 (0.47)	NA
Menstrual cycle length, d		
<21	27 565 (0.25)	NA
21-26	364 352 (3.25)	NA
27-29	6 512 288 (58.18)	NA
30-35	4 032 755 (36.03)	NA
>35	257 042 (2.30)	NA
Menstrual period length, d		
<4	3 548 893 (31.70)	NA
4-6	5 799 294 (51.81)	NA
>6	1 844 113 (16.47)	NA
Missing data	1702 (<0.01)	NA
Age at menarche, y		
<13	1 253 215 (11.20)	NA
13-14	7 119 427 (63.60)	NA
>14	2 787 828 (24.90)	NA
Missing data	33 532 (0.30)	NA
Reproductive tract infections		
Yes	187 212 (1.67)	NA
No	11 006 790 (98.33)	NA
HBsAg positive		
Yes	572 902 (5.12)	793 386 (7.10)
No	10 613 519 (94.81)	10 375 926 (92.90)
Missing data	7581 (<0.01)	24 690 (0.22)
Prior pregnancies, No.		
0	4 636 564 (41.42)	NA
≥1	6 549 205 (58.51)	NA
Missing data	8233 (<0.01)	NA

Abbreviations: BMI, body mass index (calculated as weight in kilograms divided by height in meters squared); HBsAg, hepatitis B virus surface antigen; NA, not available.

^a^ Baseline was defined as the date of the female participant’s last menstrual period.

### Preconception Levels of Serum Thyrotropin

Overall, in the fecundability analysis, the median (IQR) thyrotropin level was 1.73 (1.16-2.52) mIU/L. The 4 739 421 participants who had successfully conceived after the preconception examination had significantly lower median thyrotropin levels than those among the 6 454 581 participants who did not conceive within 1 year (eTable 4 in the Supplement). The thyrotropin levels of participants with regular menstrual cycles were significantly lower than those of participants with irregular menstrual cycles (eTable 5 in the Supplement). Among 11 194 002 included participants, 265 288 participants (2.37%) had a thyrotropin level below reference range (ie, <0.37 mIU/L), while 359 489 participants (3.21%) had a thyrotropin level above reference range (ie, >4.88 mIU/L).

### Fecundability

Within the 1-year follow-up, 4 739 421 participants became pregnant (42.34%). The pregnancy rate of group 3, the reference group, was 43.01%. The pregnancy rates of the other groups were 38.56% for group 1 (lowest thyrotropin levels), 44.30% for group 2, 40.90% for group 4, 37.82% for group 5, and 33.75% for group 6 (highest thyrotropin levels).

**Figure 2.**
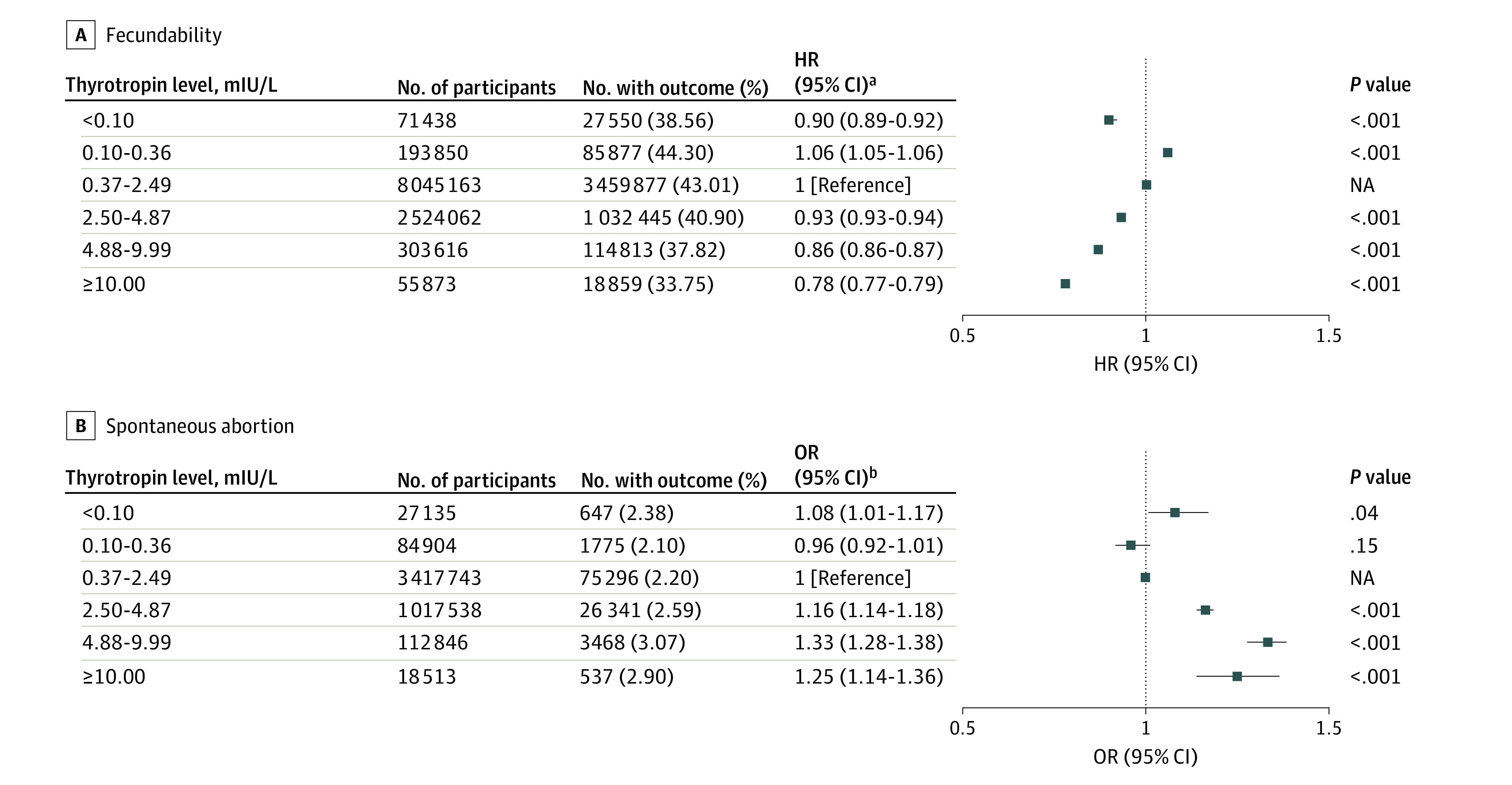
Adjusted Hazard Ratios (HRs) of Fecundability and Adjusted Odds Ratios (ORs) of Spontaneous Abortion According to Preconception Thyrotropin Levels NA indicates not applicable. ^a^Cox proportional hazard regression model was adjusted for maternal age at last menstrual period, body mass index, education, area of residence, alcohol drinking, smoking, passive smoking, hypertension, diabetes, history of thyroid disease, reproductive tract infections, hepatitis B virus surface antigen positive status, maternal age at menarche, menstrual cycle length, and menstrual period length. ^b^Logistic regression model was adjusted for maternal age at last menstrual period, body mass index, education, area of residence, alcohol drinking, smoking, passive smoking, hypertension, diabetes, history of thyroid disease, and history of adverse pregnancy outcomes.

In the multivariate analyses, compared with the reference thyrotropin group, group 1, with the lowest preconception thyrotropin levels, had lower odds of conceiving (HR, 0.90; 95% CI, 0.89-0.92), and groups with the highest thyrotropin levels also had lower odds of conception (group 5: HR, 0.86; 95% CI, 0.86-0.87; group 6, HR, 0.78; 95% CI, 0.77-0.79). However, it is noteworthy that the participants with slightly lower preconception thyrotropin levels between 0.10 and 0.36 mIU/L (group 2) had higher odds of pregnancy (HR, 1.06; 95% CI, 1.05-1.06). Detailed multivariable-adjusted HRs are listed in Figure 2; eTable 2 in the Supplement.

Similar results were observed in sensitivity analysis after the exclusion of participants with irregular menstrual cycles (eTable 6 in the Supplement) or with missing data on baseline characteristics (eTable 10 in the Supplement). In addition, the associations between preconception thyrotropin levels and TTP when stratified by gravidity were similar to the main results (eTable 8 in the Supplement).

### Risk of SA

In the cohort for SA analysis, a total of 108 064 SA events (2.31%) were documented among 4 678 679 included pregnant participants. Compared with group 3, the groups with the highest maternal preconception thyrotropin levels had higher risk of SA (group 5: OR, 1.33, 95% CI, 1.28-1.38; group 6: OR, 1.25; 95% CI, 1.14-1.36) (Figure 2; eTable 3 in the Supplement). Similar results were observed in the sensitivity analysis after excluding participants with irregular menstrual cycles (eTable 7 in the Supplement). Moreover, similar trends were obtained in participants with a history of adverse pregnancy outcomes, albeit no statistically significant associations were noted in the low (groups 1 and 2) and highest (group 6) thyrotropin levels groups (eTable 9 in the Supplement). Similar results were observed in sensitivity analysis after the exclusion of participants with missing data on baseline characteristics (eTable 11 in the Supplement).

### Dose-Response Associations

**Figure 3.**
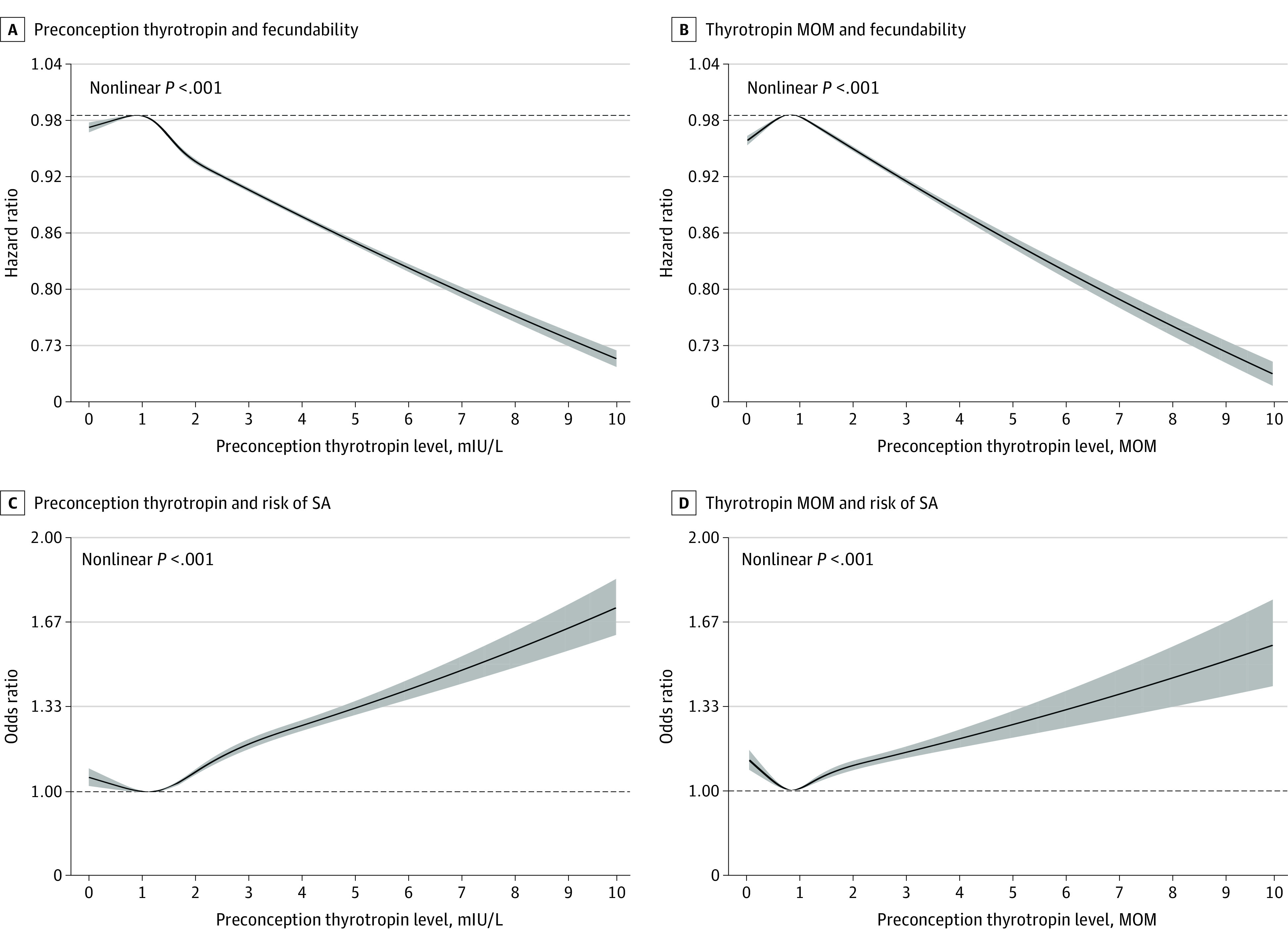
Dose-Response Association Between Maternal Preconception Thyrotropin or Thyrotropin Multiples of the Median (MOM) and Fecundability or Risk of Spontaneous Abortion (SA) A and B, Cox proportional hazard regression models were adjusted for maternal age at last menstrual period, body mass index, education, area of residence, alcohol drinking, smoking, passive smoking, hypertension, diabetes, history of thyroid disease, reproductive tract infections, hepatitis B virus surface antigen positive status, maternal age at menarche, menstrual cycle length, and menstrual period length. Reference values were 0.90 mIU/L thyrotropin (A) and 0.80 MOM thyrotropin. C and D, Logistic regression models were adjusted for maternal age at last menstrual period, body mass index, education, area of residence, alcohol drinking, smoking, passive smoking, hypertension, diabetes, history of thyroid disease, and history of adverse pregnancy outcomes. Reference values were 1.10 mIU/L thyrotropin (C) and 0.80 MOM thyrotropin (D). Black curves indicate risk estimate; shaded areas, 95% CIs.

In dose-response analyses, there was an inverted J-shaped dose-response association of preconception thyrotropin (χ^2^ = 311.29; nonlinear *P* < .001) and of thyrotropin MOM levels (χ^2^ = 510.81; nonlinear *P* < .001) with TTP (Figure 3A and B). In the restricted cubic spline analysis, we observed J-shaped dose-response associations of maternal preconception thyrotropin (χ^2^ = 58.29; nonlinear *P* < .001) and thyrotropin MOM levels (χ^2^ = 58.52; nonlinear *P* < .001, respectively) with SA (Figure 3C and D).

## Discussion

The findings of this nationwide, population-based cohort study suggest that preconception thyrotropin levels outside reference range within 1 year before pregnancy were associated with an increased risk of prolonged TTP and SA. A preconception thyrotropin level between 0.37 and 2.49 mIU/L was associated with optimal reproductive outcomes. These findings were consistent after adjusting for confounding factors and excluding participants with irregular menstrual cycles. The dose-response analysis showed an inverted J-shaped association between preconception thyrotropin level and TTP and a J-shaped association between preconception thyrotropin and risk of SA.

Few studies have investigated associations between preconception thyrotropin levels and TTP among a general population. In an Italian study of 263 participants undergoing assisted reproductive technology (ART) procedures, the clinical pregnancy rate for participants with thyrotropin levels greater than 2.5 mIU/L was lower than those with thyrotropin levels of 2.5 mIU/L or less (8.9% vs 22.3%).^24^ Consistent with our findings, a large Danish cohort of 11 254 participants suggested that elevated thyrotropin levels were associated with fewer pregnancies and live births.^25^ In contrast, in a retrospective study of 1193 participants with a history of pregnancy loss, participants with thyrotropin levels greater than 2.5 mIU/L did not have an increased risk of prolonged TTP, pregnancy loss, or live birth.^15^ The major methodological weakness of previous studies is that they enrolled participants from reproductive clinics or retrospectively gathered information from hospital databases and lacked detailed data on various relevant confounders (eg, education, BMI, and lifestyle factors). Moreover, the age and baseline thyrotropin levels of the participants from different studies were not comparable, which may affect the adjusted odds of fecundability and adverse outcomes.

Currently, there is substantial evidence on the association of overt thyroid dysfunction with adverse obstetric outcomes^26,27,28,29^; however, there is limited and inconsistent evidence on recommendations for thyroid function management in participants planning for pregnancy.^16^ In a retrospective study of 1477 participants with thyrotropin levels within reference range experiencing difficulty conceiving and undergoing ART, preconception thyrotropin levels of 2.5 to 4.99 mIU/L were associated with an increased live birth rate and decreased risk of miscarriage compared with participants with thyrotropin levels of 0.4 to 2.49 mIU/L.^14^ However, a previous population-based study within the NFPCP program including 184 611 participants recruited between 2010 and 2012 reported that participants with preconception thyrotropin levels greater than 2.5 mIU/L were more likely to experience multiple adverse pregnancy outcomes, including miscarriage, operative vaginal delivery, and preterm birth.^11^ These inconsistent associations of preconception thyrotropin levels with natural pregnancies and pregnancies achieved by ART suggest that management strategies for these 2 groups should be separated.

Our previous study among 5.8 million participants in China who subsequently became pregnant found that preconception thyrotropin levels outside reference range were significantly associated with increased risk of preterm birth, small for gestational age, and perinatal infant death.^12^ However, at that time, we were unable to evaluate outcomes regarding fecundability or miscarriage owing to lack of data on these outcomes. In this study, we also found a J-shape association of preconception thyrotropin levels with the risk of SA. In terms of hyperthyroidism, our findings suggest that participants with thyrotropin levels less than 0.1 mIU/L had an increased risk of SA, while mild suppression of thyrotropin was not associated with risk of SA. Given the large number of participants and the absence of medication history in this study, practitioners should be cautious when interpreting the clinical relevance of hyperthyroidism for reproductive outcomes.

Notably, our study found that compared with women with thyrotropin levels of 4 to 10 mIU/L, women with thyrotropin greater than 10 mIU/L were more likely to have delayed TTP, but they experienced a lower risk of pregnancy loss. This corroborates with our previous study on the association between preconception thyrotropin and adverse pregnancy outcomes.^12^ These associations might exist because overt hypothyroidism is more likely to be treated by clinicians, while a standardized management strategy for SCH is absent. These findings reveal the current dilemma of SCH treatment and the continuous association of thyrotropin levels outside reference range with adverse pregnancy outcomes.^30^

Universal screening for thyroid dysfunction before or during pregnancy has been controversial. Findings of a systemic review^31^ of 2 randomized clinical trials suggest that universal screening of thyroid function in early pregnancy was not associated with improved pregnancy outcomes or fetal cognitive function. Additionally, a study from the US^32^ reported that the treatment of maternal SCH was not significantly associated with improved fetal cognitive outcomes. As thyroid hormone has a time-dependent role on placental and fetal development,^5,7^ it is possible that the time of treatment initiation in previous trials^32,33,34^ (8th-20th gestation week) missed the optimal intervention time window. An evolving body of evidence has demonstrated associations between preconception thyrotropin levels outside reference range and adverse pregnancy outcomes^11,12^ and shown the benefits associated with levothyroxine treatment among periconception participants with SCH.^30,35^ Therefore, there are calls by endocrine societies for screening and early management of thyroid dysfunction for patients with pregnancy plans. However, with uncertainties about the interpretation of screening results in specific subpopulations and the lack of evidence on the benefits of levothyroxine treatment among individuals planning for pregnancy, it is currently difficult for clinicians to decide the appropriate circumstances to initiate treatment. Focusing on a more generalized population without diagnosed infertility, our study found that preconception thyrotropin levels outside reference range were associated with increased risk of reduced fecundability and of miscarriage, with the highest risk among participants with thyrotropin levels of 4.88 to 9.99 mIU/L. Our findings provide insights for the implementation of preconception thyroid function screening and the design of future levothyroxine supplementation trials.

To our knowledge, this is the first study investigating the associations of preconception thyrotropin with TTP and pregnancy loss in a large population-based cohort. An important strength of our study is the scale of participant enrolment from 31 provinces across China. The design of the NFPCP allowed us to focus on 2 end points (TTP and SA) in a large consecutively enrolled cohort study. Also, the detailed information on individual covariates provided adequate statistical power for the subgroup and sensitivity analysis. Our exclusion of participants with diagnosed infertility and application of thyrotropin MOM improved the generalizability for preconception counselling and health care policy.

### Limitations

This study has several limitations. First, in dearth of data about thyroxine and thyroid autoantibody concentrations, the evaluation of thyroid function status was solely based on thyrotropin levels, which may lead to misclassification. This is an important limitation, as the extent of thyroid dysfunction and the status of thyroid autoimmunity have different associations with pregnancy outcomes.^7^ However, in consideration of staffing and material resources, it is impractical to perform detailed thyroid function tests for all participants. Second, maternal thyrotropin levels and thyroid hormones fluctuated throughout gestation,^16^ but thyrotropin levels were measured only once before pregnancy. It is possible that some participants with thyroid dysfunction received thyroid medication during gestation, which may underestimate the associations. To compensate for this limitation, we adjusted for thyroid disease history in our regression model, and the results were consistent with our main findings. Third, although the conception status was identified by urinary pregnancy test and ultrasonographic diagnosis in local maternal and child health hospital, it is possible that our acquisition of pregnancy outcome data may have been biased, given that participants who experienced SA might have been reluctant to report this pregnancy outcome or even misidentified SA events as delayed menstruation. Fourth, we did not collect information about couples’ fertility, such as intercourse frequency or sperm count or concentration, although this study had strict exclusion criteria and adjusted many covariates on fertility. Fifth, although we were able to adjust for many cofounding factors in multivariable analysis, our study estimates did not account for unknown and unmeasured residual confounding factors. Previous studies reported that iodine deficiency was associated with miscarriage and infant death^36^; however, we did not stratify the participants according to their iodine status since the relevant data were lacking. Nonetheless, China has implemented the universal salt iodization policy, and results from a national survey indicate that most areas are iodine-sufficient.^37^ Moreover, since the NFPCP only included Han Chinese participants, the results in our study may be not generalizable to other ethnic groups. In brief, the observational design of our study is unable to draw causal associations.

## Conclusions

To our knowledge, this is the first population-based cohort study to report that preconception thyrotropin levels outside reference range may be associated with reduced fecundability and increased risk of SA. Our findings support that a preconception thyrotropin between the lower reference limit and 2.5 mIU/L was associated with the lowest risk of these adverse reproductive outcomes. These findings provide important evidence for preconception health care and suggest that interventional studies investigating the benefits of preconception thyroid function screening and levothyroxine supplementation are warranted.

## Notes

Supplement. **eAppendix 1.** Supplemental Methods **eAppendix 2.** Variables Definitions **eTable 1.** Characteristics of the Total Study Population for Spontaneous Abortion Analysis **eTable 2.** Adjusted Hazard Ratios (HRs) of Fecundability Associated With Preconception Thyrotropin Levels of Participants **eTable 3.** Adjusted Odds Ratios (ORs) of Spontaneous Abortion According to Preconception Thyrotropin Levels of Participants **eTable 4.** The Differences of Thyrotropin Levels of Participants According to Pregnancy Status Within 1 Year **eTable 5.** The Differences of Thyrotropin Levels of Participants According to Menstrual Cycle **eTable 6.** Adjusted Hazard Ratios (HRs) of Fecundability Associated With Preconception Thyrotropin Levels of Participants After Excluding Participants With Data on Irregular Menstrual Cycles **eTable 7.** Adjusted Odds Ratios (ORs) of Spontaneous Abortion According to Preconception Thyrotropin Levels of Participants After Excluding Participants With Data on Irregular Menstrual Cycles **eTable 8.** Adjusted Hazard Ratios (HRs) of Fecundability Associated With Preconception Thyrotropin Levels of Participants According to Gravidity **eTable 9.** Adjusted Odds Ratios (ORs) of Spontaneous Abortion Associated With Preconception Thyrotropin Levels of Participants According to History of Adverse Pregnancy Outcomes **eTable 10.** Adjusted Hazard Ratios (HRs) of Fecundability Associated With Preconception Thyrotropin Levels of Participants After Excluding Participants With Missing Data on Baseline Characteristics **eTable 11.** Adjusted Odds Ratios (ORs) of Spontaneous Abortion According to Preconception Thyrotropin Levels of Participants After Excluding Participants With Missing Data on Baseline Characteristics
