# Supplementary material for: Association of Preconception Thyrotropin Levels With Fecundability and Risk of Spontaneous Abortion in China
Source: JAMA Netw Open. 2022 Aug 31;5(8):e2228892. doi: 10.1001/jamanetworkopen.2022.28892 (PMC9434356; doi:10.1001/jamanetworkopen.2022.28892)
Supplement: Supplement. — eAppendix 1. Supplemental Methods eAppendix 2. Variables Definitions eTable 1. Characteristics of the Total Study Population for Spontaneous Abortion Analysis eTable 2. Adjusted Hazard Ratios (HRs) of Fecundability Associated With Preconception Thyrotropin Levels of Participants eTable 3. Adjusted Odds Ratios (ORs) of Spontaneous Abortion According to Preconception Thyrotropin Levels of Participants eTable 4. The Differences of Thyrotropin Levels of Participants According to Pregnancy Status Within 1 Year eTable 5. The Differences of Thyrotropin Levels of Participants According to Menstrual Cycle eTable 6. Adjusted Hazard Ratios (HRs) of Fecundability Associated With Preconception Thyrotropin Levels of Participants After Excluding Participants With Data on Irregular Menstrual Cycles eTable 7. Adjusted Odds Ratios (ORs) of Spontaneous Abortion According to Preconception Thyrotropin Levels of Participants After Excluding Participants With Data on Irregular Menstrual Cycles eTable 8. Adjusted Hazard Ratios (HRs) of Fecundability Associated With Preconception Thyrotropin Levels of Participants According to Gravidity eTable 9. Adjusted Odds Ratios (ORs) of Spontaneous Abortion Associated With Preconception Thyrotropin Levels of Participants According to History of Adverse Pregnancy Outcomes eTable 10. Adjusted Hazard Ratios (HRs) of Fecundability Associated With Preconception Thyrotropin Levels of Participants After Excluding Participants With Missing Data on Baseline Characteristics eTable 11. Adjusted Odds Ratios (ORs) of Spontaneous Abortion According to Preconception Thyrotropin Levels of Participants After Excluding Participants With Missing Data on Baseline Characteristics [file jamanetwopen-e2228892-s001.pdf]

Supplemental Online Content

Yang Y, Guo T, Fu J, et al. Association of preconception thyrotropin levels with fecundability and risk of spontaneous abortion in China. *JAMA Netw Open*. 2022;5(8):e2228892. doi:10.1001/jamanetworkopen.2022.28892

eAppendix 1. Supplemental Methods
eAppendix 2. Variables Definitions
eTable 1. Characteristics of the Total Study Population for Spontaneous Abortion Analysis
eTable 2. Adjusted Hazard Ratios (HRs) of Fecundability Associated With Preconception Thyrotropin Levels of Participants
eTable 3. Adjusted Odds Ratios (ORs) of Spontaneous Abortion According to Preconception Thyrotropin Levels of Participants
eTable 4. The Differences of Thyrotropin Levels of Participants According to Pregnancy Status Within 1 Year
eTable 5. The Differences of Thyrotropin Levels of Participants According to Menstrual Cycle
eTable 6. Adjusted Hazard Ratios (HRs) of Fecundability Associated With Preconception Thyrotropin Levels of Participants After Excluding Participants With Data on Irregular Menstrual Cycles
eTable 7. Adjusted Odds Ratios (ORs) of Spontaneous Abortion According to Preconception Thyrotropin Levels of Participants After Excluding Participants With Data on Irregular Menstrual Cycles
eTable 8. Adjusted Hazard Ratios (HRs) of Fecundability Associated With Preconception Thyrotropin Levels of Participants According to Gravidity
eTable 9. Adjusted Odds Ratios (ORs) of Spontaneous Abortion Associated With Preconception Thyrotropin Levels of Participants According to History of Adverse Pregnancy Outcomes
eTable 10. Adjusted Hazard Ratios (HRs) of Fecundability Associated With Preconception Thyrotropin Levels of Participants After Excluding Participants With Missing Data on Baseline Characteristics
eTable 11. Adjusted Odds Ratios (ORs) of Spontaneous Abortion According to Preconception Thyrotropin Levels of Participants After Excluding Participants With Missing Data on Baseline Characteristics

This supplemental material has been provided by the authors to give readers additional information about their work.

© 2022 Yang Y et al. *JAMA Network Open.*

eAppendix 1. Supplemental Methods

In the first stage, couples who met the fertility policy and planned to conceive within 6 months, were advocated and encouraged by the local resident committee to participate in a preconception health examination in local maternal and child service centers. Preconception blood samples were collected at local maternal and child service centers to measure thyroid stimulating hormone, fasting plasma glucose (only for female participants) and hepatitis B virus surface antigen (HBsAg) status after at least 8 hours of fasting. In addition, participant's demographics, family history, past medication and disease history (including congenital malformations history), dietary nutrition, lifestyle, environmental poisons, social-psychological factors, clinical examination and physical examination were recorded in the preconception medical chart. All participants underwent reproductive system examinations, and the female participants additionally to undergo ultrasound imaging to rule out reproductive organ malformations or other abnormalities that affected fertility. Furthermore, the female participants' secretions, which were microscopically examined for leucorrhea including routine leucorrhea examination items Gardnerella vaginalis, Candida, Neisseria gonorrhoeae, Trichomonas vaginalis or Chlamydia trachomatis infections, were obtained by vaginal swabs.

In the second stage, early pregnancy follow-up interview was conducted by trained staff using telephone, within 3 months after the preconception health checkups stage completed, to obtain the conception status of eligible participants. If the participants did not get pregnant at the first follow-up interview, repeated inquiries were conducted subsequently within the next three months until 1 year after preconception health checkups. Women who self-reported as pregnant were required to have their pregnancies confirmed by doctors and to undergo ultrasound imaging. The latter was used to calculate the first day of the last menstrual period (LMP). The ultrasound-adjusted LMP and information on toxic or harmful substances exposure, and any lifestyle changes during the first trimester of pregnancy were collected in this stage, The National Center of Clinical Laboratories for Quality Inspection and Detection was responsible for quality evaluation to ensure the accuracy and reliability of inspection data.

In the third stage, the study participants who had become pregnant by the time of the first follow-up survey were recontacted by the local trained interviewers, within 1 year after early pregnancy follow-up, to collect pregnancy outcome information, in which abortion, induced labor, still birth, birth defects, PTB, low birth weight, and ectopic pregnancy were documented as adverse pregnancy outcomes. A pregnancy outcome follow-up interview will be conducted by research staffs through telephone active report from participants during early pregnancy period, if the participants had abortion or other problems, especially the adverse pregnancy outcome. In addition, delivery condition of the mother and vital newborn information such as the delivery date, delivery mode, and birth weight, were also obtained from puerpera's self-reported information. The gestational week was calculated by the duration between the ultrasound-adjusted LMP and the date of delivery.

© 2022 Yang Y et al. *JAMA Network Open.*

eAppendix 2. Variables Definition

The ages of participants were calculated as the difference between the date of birth and the first day of the LMP of participants; these ages were categorized into various age groups (20–24.9, 25–29.9, 30–34.9, 35–39.9, and ≥ 40 years). The body weight and height of wives and their husbands wearing light, indoor clothing and no shoes were measured according to a standardized protocol. Next, BMI was calculated. Preconception body mass index (BMI) were categorized into various groups (<18.5 kg/m2 , 18.5-23.9 kg/m2 , 24.0-27.9 kg/m2 , and ≥28.0 kg/m2).Higher education was categorized as levels of education of senior high school, college or higher. Diabetes mellitus was categorized as either self-reported diabetes or fasting blood glucose ≥7.0mmol/L (Yes, No). Diabetes mellitus for male participants was categorized as self-reported diabetes (Yes, No). (Fasting blood glucose were not tested in the male participants.) Hypertension was defined as self-reported hypertension or systolic blood pressure ≥140 mmHg or diastolic blood pressure ≥90 mmHg (Yes, No). Smoking was defined as smoking before or during early pregnancy (participants who smoked at least 1 cigarette per day at least one year at the time of baseline examination) (Yes, No). Passive smoking was defined as exposure to environmental tobacco smoke before or during early pregnancy (Yes, No). Alcohol drinking was defined as drinking before or during early pregnancy (Yes, No). Area of residence was divided into 2 categories: rural or urban. Number of pregnancies (gravidity) was divided into 2 categories: =0 or ≥1. Positivity of hepatitis B virus surface antigen testing (negative, positive). Thyroid disease history was based on self-report, including hyperthyroidism, Hashimoto's thyroiditis, goitre, thyroid cancers and so on (Yes, No). Genital tract infection was defined as a female partner was infected by at least one of the following pathogens: Candida, Trichomonas vaginalis, Chlamydia trachomatis, Gardnerella vaginalis or Neisseria gonorrhoeae. According to the interquartile interval, age at menarche was divided into <13, 13–14, and >14 years old, and the menstrual period length was divided into <4, 4–6, and >6 days, respectively. The menstrual cycle length was divided into <21, 21–26, 27–29, 30–35 and >35 days. History of adverse pregnancy outcomes was defined as the history of preterm birth, later fetal death or spontaneous abortion in previous pregnancies.

© 2022 Yang Y et al. *JAMA Network Open.*

eTable 1. Characteristics of the Total Study Population for Spontaneous Abortion Analysis[a]

characteristics		Maternal characteristics (n=4 678 679)
Age at LMP (years)		
20-24.9		1 900 376 (40.62)
25-29.9		1 959 033 (41.87)
30-34.9		590 914 (12.63)
35-39.9		191 146 (4.09)
≥40		37 210 (0.80)
Missing data		NA
BMI (kg/m²)		
Underweight (<18.5)		604 555 (12.92)
Normal weight (18.5-)		3 343 031 (71.45)
Overweight (24.0-)		583 066 (12.46)
Obese (28.0-)		136 526 (2.92)
Missing data		11 501 (0.25)
Thyrotropin level (mIU/L)		
<0.36		112 039 (2.39)
0.36-4.87		4 435 281 (94.80)
≥4.88		131 359 (2.81)
Education		
High school or above		821 742 (17.56)
Primary school or below		3 777 664 (80.74)
Missing data		79 273 (1.69)
Residence		
Rural		4 282 221 (91.53)
Urban		369 213 (7.89)
Missing data		245 (<0.01)
Alcohol consumption		
Yes		114 638 (2.45)
No		4 551 554 (97.28)
Missing data		12 487 (0.27)
Smoking status		
Yes		8 683 (0.19)
No		4 661 240 (99.63)
Missing data		8 756 (0.19)
Second-hand smoke		
Yes		1 055 281 (22.56)
No		3 610 308 (77.17)
Missing data		8 999 (0.19)
Hypertension		
Yes		75 577 (1.62)

© 2022 Yang Y et al. *JAMA Network Open.*

No		4 582 184 (97.94)
Missing data		20 918 (0.45)
Diabetes mellitus		
Yes		45 832 (0.98)
No		4 612 137 (98.58)
Missing data		20 710 (0.44)
Gravidity		
0		2 469 578 (52.78)
≥1		2 205 341 (47.14)
Missing data		3 760 (0.08)
History of adverse pregnancy outcomes		
Yes		734 031 (15.69)
No		3 926 591 (83.93)
Missing data		18 057 (0.39)

[a] Data presented as number (percentage) unless otherwise indicated.

Abbreviations: LMP, last menstrual period; BMI, body mass index (calculated as the weight in kilograms divided by height in meters squared); NA, not available.

© 2022 Yang Y et al. *JAMA Network Open.*

eTable 2 Adjusted Hazard Ratios (HRs) of Fecundability Associated With Preconception Thyrotropin Levels of Participants

	No. of participants	Pregnant participants n (%)	TTP* median (IQR**)	Model 1[a] HR (95% CI)	Model 2[b] HR (95% CI)
**Thyrotropin level (mIU/L)**					
<0.10	71 438	27 550 (38.56)	6.00 (8.70)	0.88 (0.87-0.89)	0.90 (0.89-0.92)
0.10-0.36	193 850	85 877 (44.30)	5.30 (8.90)	1.05 (1.04-1.06)	1.06 (1.05-1.06)
0.37-2.49	8 045 163	3 459 877 (43.01)	5.60 (8.70)	1 [Reference]	1 [Reference]
2.50-4.87	2 524 062	1 032 445 (40.90)	6.06 (8.58)	0.93 (0.93-0.94)	0.93 (0.93-0.94)
4.88-9.99	303 616	114 813 (37.82)	6.54 (8.33)	0.85 (0.84-0.85)	0.86 (0.86-0.87)
≥10.00	55 873	18 859 (33.75)	6.67 (8.07)	0.76 (0.75-0.77)	0.78 (0.77-0.79)

[a] Model 1 was adjusted for maternal age at last menstrual period.

[b] Model 2 was adjusted for maternal age at last menstrual period, body mass index, education, area of residence, alcohol drinking, smoking, passive smoking, hypertension, diabetes, history of thyroid disease, reproductive tract infections, hepatitis B virus surface antigen positive status, maternal age at menarche, menstrual cycle length, menstrual period length.

Abbreviations: *TTP, time to pregnancy; **IQR, interquartile range.

© 2022 Yang Y et al. *JAMA Network Open.*

eTable 3. Adjusted Odds Ratios (ORs) of Spontaneous Abortion According to Preconception Thyrotropin Levels of Participants

	No. of	Spontaneous abortion	Model 1[a]	Model 2[b]
	**participants**	**n (%)**	**OR (95% CI)**	**OR (95% CI)**
**Thyrotropin level (mIU/L)**				
<0.10	27 135	647 (2.38)	1.09 (1.01-1.18)	1.08 (1.01-1.17)
0.10-0.36	84 904	1 775 (2.10)	0.96 (0.92-1.01)	0.96 (0.92-1.01)
0.37-2.49	3 417 743	75 296 (2.20)	1 [Reference]	1 [Reference]
2.50-4.87	1 017 538	26 341 (2.59)	1.17 (1.15-1.18)	1.16 (1.14-1.18)
4.88-9.99	112 846	3 468 (3.07)	1.37 (1.32-1.42)	1.33 (1.28-1.38)
≥10.00	18 513	537 (2.90)	1.28 (1.17-1.39)	1.25 (1.14-1.36)

[a] Model 1 was adjusted for maternal age at last menstrual period.

[b] Model 2 was adjusted for maternal age at last menstrual period, body mass index, education, area of residence, alcohol drinking, smoking, passive smoking, hypertension, diabetes, history of thyroid disease, history of adverse pregnancy outcomes.

© 2022 Yang Y et al. *JAMA Network Open.*

eTable 4. The Differences of Thyrotropin Levels of Participants According to Pregnancy Status Within 1 Year

	Pregnant participants		Unpregnant participants		
**Thyrotropin level (mIU/L)**	**n (%)**	**median (IQR[a])**	**n (%)**	**median (IQR)**	**P[b]**
	4 739 421 (42.34)	1.70 (1.33)	6 454 581 (57.66)	1.75 (1.39)	<0.001
**<0·37 mIU/L**	113 427 (2.39)	0.23 (0.21)	151 861 (2.35)	0.22 (0.24)	<0.001
**0·37-4·88 mIU/L**	4 492 322 (94.78)	1.69 (1.23)	6 076 903 (94.14)	1.74 (1.27)	<0.001
**≥4·88 mIU/L**	133 672 (2.82)	5.95 (2.36)	225 817 (3.50)	6.09 (2.71)	<0.001

Abbreviations: [a] IQR, interquartile range.

[b] The Mann-Whitney U-test was used to examine the differences of thyrotropin levels among two groups (Pregnant group v. Unpregnant group).

© 2022 Yang Y et al. *JAMA Network Open.*

eTable 5. The Differences of Thyrotropin Levels of Participants According to Menstrual Cycle

	Regular menstrual cycle		Irregular menstrual cycle		
**Thyrotropin level (mIU/L)**	**n (%)**	**median (IQR[a])**	**n (%)**	**median (IQR)**	**P[b]**
	10 859 029 (97.01)	1.73 (1.36)	334 973 (2.99)	1.80 (1.40)	<0.001
**<0·37 mIU/L**	257 539 (2.37)	0.23 (0.23)	7 749 (2.31)	0.20 (0.25)	<0.001
**0·37-4·88 mIU/L**	10 254 982 (94.44)	1.71 (1.26)	314 243 (93.81)	1.77 (1.29)	<0.001
**≥4·88 mIU/L**	346 508 (3.19)	6.03 (2.58)	12 981 (3.87)	6.10 (2.62)	0.002

Abbreviations: [a] IQR, interquartile range.

[b] The Mann-Whitney U-test was used to examine the differences of thyrotropin levels among two groups (Regular menstrual cycle group v. Irregular menstrual cycle group).

© 2022 Yang Y et al. *JAMA Network Open.*

eTable 6. Adjusted Hazard Ratios (HRs) of Fecundability Associated With Preconception Thyrotropin Levels of Participants After Excluding Participants With Data on Irregular Menstrual Cycles

	**No. of**	**Pregnant participants**	**Model 1[a]**	**Model 2[b]**
	**participants**	**n (%)**	**HR (95% CI)**	**HR (95% CI)**
**Thyrotropin level (mIU/L)**				
<0.10	68 921	26 677 (38.71)	0.88 (0.87-0.89)	0.90 (0.89-0.92)
0.10-0.36	188 618	83 602 (44.32)	1.04 (1.03-1.05)	1.05 (1.04-1.06)
0.37-2.49	7 810 804	3 370 653 (43.15)	1 [Reference]	1 [Reference]
2.50-4.87	2 444 178	1 002 655 (41.02)	0.93 (0.93-0.94)	0.93 (0.93-0.94)
4.88-9.99	292 638	110 916 (37.90)	0.85 (0.84-0.85)	0.86 (0.86-0.87)
≥10.00	53 870	18 227 (33.84)	0.76 (0.75-0.77)	0.78 (0.77-0.79)

[a] Model 1 was adjusted for maternal age at last menstrual period.

[b] Model 2 was adjusted for maternal age at last menstrual period, body mass index, education, area of residence, alcohol drinking, smoking, passive smoking, hypertension, diabetes, history of thyroid disease, reproductive tract infections, hepatitis B virus surface antigen positive status, maternal age at menarche, menstrual cycle length, menstrual period length.

© 2022 Yang Y et al. *JAMA Network Open.*

eTable 7. Adjusted Odds Ratios (ORs) of Spontaneous Abortion According to Preconception Thyrotropin Levels of Participants After Excluding Participants With Data on Irregular Menstrual Cycles

	**No. of**	**Spontaneous abortion**	**Model 1[a]**	**Model 2[b]**
	**participants**	**n (%)**	**OR (95% CI)**	**OR (95% CI)**
**Thyrotropin level (mIU/L)**				
<0.10	26 272	627 (2.39)	1.10 (1.01-1.19)	1.09 (1.01-1.18)
0.10-0.36	82 653	1 713 (2.07)	0.96 (0.92-1.01)	0.96 (0.91-1.01)
0.37-2.49	3 329 957	72 940 (2.19)	1 [Reference]	1 [Reference]
2.50-4.87	988 298	25 480 (2.58)	1.17 (1.15-1.19)	1.16 (1.14-1.18)
4.88-9.99	109 009	3 342 (3.07)	1.37 (1.33-1.42)	1.33 (1.29-1.38)
≥10.00	17 892	518 (2.90)	1.28 (1.17-1.40)	1.25 (1.14-1.36)

[a] Model 1 was adjusted for maternal age at last menstrual period.

[b] Model 2 was adjusted for maternal age at last menstrual period, body mass index, education, area of residence, alcohol drinking, smoking, passive smoking, hypertension, diabetes, history of thyroid disease, history of adverse pregnancy outcomes.

© 2022 Yang Y et al. *JAMA Network Open.*

eTable 8. Adjusted Hazard Ratios (HRs) of Fecundability Associated With Preconception Thyrotropin Levels of Participants According to Gravidity

	No. of	Pregnant participants	Model 1[a]	Model 2[b]
	participants	n (%)	HR (95% CI)	HR (95% CI)
**Thyrotropin level (mIU/L)**				
**Gravidity (=0)**	4 636 564			
<0.10	28 468	14 242 (50.03)	0.91 (0.90-0.93)	0.93 (0.92-0.95)
0.10-0.36	82 552	46 169 (55.93)	1.08 (1.07-1.09)	1.07 (1.06-1.08)
0.37-2.49	3 381 219	1 840 483 (54.43)	1 [Reference]	1 [Reference]
2.50-4.87	1 015 620	527 751 (51.37)	0.92 (0.91-0.92)	0.93 (0.92-0.93)
4.88-9.99	110 353	53 653 (48.62)	0.83 (0.82-0.83)	0.84 (0.83-0.85)
≥10.00	18 352	8 299 (45.22)	0.76 (0.75-0.78)	0.77 (0.75-0.79)
**Gravidity (≥1)**	6 549 205			
<0.10	42 914	13 276 (30.94)	0.88 (0.86-0.89)	0.88 (0.87-0.90)
0.10-0.36	111 161	39 643 (35.66)	1.03 (1.02-1.04)	1.04 (1.03-1.05)
0.37-2.49	4 657 976	1 616 600 (34.71)	1 [Reference]	1 [Reference]
2.50-4.87	1 506 641	503 885 (41.02)	0.95 (0.94-0.95)	0.95 (0.94-0.95)
4.88-9.99	193 035	61 081 (33.44)	0.89 (0.88-0.90)	0.89 (0.89-0.90)
≥10.00	37 478	10 539 (28.12)	0.79 (0.78-0.81)	0.80 (0.78-0.81)

[a] Model 1 was adjusted for maternal age at last menstrual period.

[b] Model 2 was adjusted for maternal age at last menstrual period, body mass index, education, area of residence, alcohol drinking, smoking, passive smoking, hypertension, diabetes, history of thyroid disease, reproductive tract infections, hepatitis B virus surface antigen positive status, maternal age at menarche, menstrual cycle length, menstrual period length.

© 2022 Yang Y et al. *JAMA Network Open.*

eTable 9. Adjusted Odds Ratios (ORs) of Spontaneous Abortion Associated With Preconception Thyrotropin Levels of Participants According to History of Adverse Pregnancy Outcomes

	No. of participants	Spontaneous abortion n (%)	Model 1[a] OR (95% CI)	Model 2[b] OR (95% CI)
**Thyrotropin level (mIU/L)**				
**No history of adverse pregnancy outcomes**	3 926 591			
<0.10	21 958	479 (2.23)	1.09 (0.99-1.19)	1.09 (0.99-1.19)
0.10-0.36	71 009	1 375 (1.94)	0.97 (0.92-1.02)	0.98 (0.92-1.03)
0.37-2.49	2 887 678	58 364 (2.03)	1 [Reference]	1 [Reference]
2.50-4.87	842 209	20 106 (2.40)	1.18 (1.16-1.20)	1.17 (1.15-1.19)
4.88-9.99	88 997	2 523 (2.84)	1.39 (1.33-1.44)	1.37 (1.31-1.43)
≥10.00	14 740	406 (2.71)	1.33 (1.21-1.47)	1.31 (1.19-1.45)
**History of adverse pregnancy outcomes (≥1)**	734 031			
<0.10	5 087	167 (3.26)	1.04 (0.89-1.21)	1.05 (0.90-1.23)
0.10-0.36	13 669	399 (2.96)	0.93 (0.84-1.03)	0.92 (0.83-1.02)
0.37-2.49	517 848	16 743 (3.24)	1 [Reference]	1 [Reference]
2.50-4.87	170 455	6 151 (3.62)	1.11 (1.08-1.15)	1.11 (1.08-1.15)
4.88-9.99	23 273	937 (4.06)	1.25 (1.16-1.33)	1.23 (1.15-1.32)
≥10.00	3 699	130 (3.51)	1.07 (0.89-1.27)	1.07 (0.89-1.27)

[a] Model 1 was adjusted for maternal age at last menstrual period.

[b] Model 2 was adjusted for maternal age at last menstrual period, body mass index, education, area of residence, alcohol drinking, smoking, passive smoking, hypertension, diabetes, history of thyroid disease, history of adverse pregnancy outcomes.

© 2022 Yang Y et al. *JAMA Network Open.*

eTable 10. Adjusted Hazard Ratios (HRs) of Fecundability Associated With Preconception Thyrotropin Levels of Participants After Excluding Participants With Missing Data on Baseline Characteristics

	**No. of**	**Pregnant participants**	**Model 1[a]**	**Model 2[b]**
	**participants**	**n (%)**	**HR (95% CI)**	**HR (95% CI)**
**Thyrotropin level (mIU/L)**				
<0.10	67 200	26 017 (38.71)	0.88 (0.87-0.89)	0.91 (0.89-0.92)
0.10-0.36	184 148	81 879 (44.46)	1.05 (1.04-1.06)	1.06 (1.05-1.06)
0.37-2.49	7 643 811	3 297 376 (43.14)	1 [Reference]	1 [Reference]
2.50-4.87	2 395 655	982 741 (41.02)	0.93 (0.93-0.94)	0.93 (0.93-0.94)
4.88-9.99	286 867	108 761 (37.91)	0.85 (0.84-0.85)	0.86 (0.86-0.87)
≥10.00	52 713	17 841(33.85)	0.76 (0.74-0.77)	0.78 (0.77-0.79)

[a] Model 1 was adjusted for maternal age at last menstrual period.

[b] Model 2 was adjusted for maternal age at last menstrual period, body mass index, education, area of residence, alcohol drinking, smoking, passive smoking, hypertension, diabetes, history of thyroid disease, reproductive tract infections, hepatitis B virus surface antigen positive status, maternal age at menarche, menstrual cycle length, menstrual period length.

© 2022 Yang Y et al. *JAMA Network Open.*

eTable 11. Adjusted Odds Ratios (ORs) of Spontaneous Abortion According to Preconception Thyrotropin Levels of Participants After Excluding Participants With Missing Data on Baseline Characteristics

	No. of	Spontaneous abortion	Model 1[a]	Model 2[b]
	participants	n (%)	OR (95% CI)	OR (95% CI)
**Thyrotropin level (mIU/L)**				
<0.10	25 880	629 (2.43)	1.11 (1.03-1.21)	1.08 (1.00-1.17)
0.10-0.36	81 826	1 706 (2.08)	0.96 (0.92-1.01)	0.96 (0.91-1.01)
0.37-2.49	3 291 614	72 461 (2.20)	1 [Reference]	1 [Reference]
2.50-4.87	978 081	25 423 (2.60)	1.17 (1.16-1.19)	1.16 (1.14-1.18)
4.88-9.99	108 002	3 335 (3.08)	1.38 (1.33-1.43)	1.33 (1.28-1.38)
≥10.00	17 672	516 (2.92)	1.29 (1.18-1.41)	1.24 (1.14-1.36)

[a] Model 1 was adjusted for maternal age at last menstrual period.

[b] Model 2 was adjusted for maternal age at last menstrual period, body mass index, education, area of residence, alcohol drinking, smoking, passive smoking, hypertension, diabetes, history of thyroid disease, history of adverse pregnancy outcomes, history of adverse pregnancy outcomes.

© 2022 Yang Y et al. *JAMA Network Open.*
